# G Protein α Subunit GpaB is Required for Asexual Development, Aflatoxin Biosynthesis and Pathogenicity by Regulating cAMP Signaling in *Aspergillus flavus*

**DOI:** 10.3390/toxins10030117

**Published:** 2018-03-10

**Authors:** Yinghang Liu, Kunlong Yang, Qiuping Qin, Guinan Lin, Tianran Hu, Zhangling Xu, Shihua Wang

**Affiliations:** 1 Fujian Key Laboratory of Pathogenic Fungi and Mycotoxins, Key Laboratory of Biopesticide and Chemical Biology of Education Ministry, and School of Life Sciences, Fujian Agriculture and Forestry University, Fuzhou 350002, China; lyinghang35324@163.com (Y.L.); haroldqin@aliyun.com (Q.Q.); linguinan1996@outlook.com (G.L.); hutianran77@yeah.net (T.H.); xuzhangling0409@163.com (Z.X.); 2 Xiamen Anjie Medical Data Technology Co. Ltd., Xiamen 361115, China

**Keywords:** G protein, sclerotia, fungal virulence, cAMP

## Abstract

The heterotrimeric G proteins are critical for signal transduction and function in numerous biological processes including vegetative growth, asexual development and fungal virulence in fungi. Here, we identified four G protein alpha subunits (GanA, GpaB, FadA and GaoC) in the notorious Aflatoxin-producing fungus *Aspergillus flavus*. GanA, GpaB and FadA have homologues in other fungal species, while GaoC is a novel one. Here, we showed that the loss function of *gpaB* displayed a defect in conidiophore formation and considerably reduced expression levels of conidia-specific genes *brlA* and *abaA*. A decreased viability of cell wall integrity stress and oxidative stress were also found in the ∆*gpaB* mutant. More importantly, aflatoxin (AF) biosynthesis and infection on crop seeds were severely impaired in the *gpaB*-deficient mutant. Further analyses demonstrated that the intracellular cAMP levels significantly reduced in the *gpaB*-deficient mutant compared to wildtype strains. Additionally, an alteration of PKA activities in the ∆*gpaB* mutant was also found. Overall, our results indicated that GpaB played diverse roles in asexual sporulation, AF biosynthesis and virulence by regulating cAMP signaling in *Aspergillus flavus*.

## 1. Introduction

Aflatoxins (AFs) are ranked as one of the most toxic carcinogens for human and animal known in nature. The AFs producing fungus *Aspergillus flavus* contaminates several important seed crops and food stuffs. Under favorable conditions, *A. flavus* challenged crop seeds, accumulating the toxic secondary metabolites [1], which has caused serious agriculture problems and posed a threat to human health. Therefore, it is quite important to control the contamination of this fungus on the crops both at pre- and post-harvest stages.

Accumulating studies have demonstrated that the pathogenicity of *A. flavus* has a close relationship with fungal growth, mycotoxins and the adaptability to environmental stresses [1,2]. In fungi, the G protein signaling, which is a key element of signal transduction pathways, engages in the regulation of a range of physiological processes, including fungal development and virulence, in response to environmental stimuli [3,4,5]. In eukaryotes, each G-protein is composed of α, β and γ subunits, of which β and γ subunits interact as a heterodimer [6]. The heterotrimeric complex, binding with GDP, is associated with the G protein-coupled receptor (GPCR) in the inactive state. Once activated, the exchanging of GTP for GDP happens on the Gα protein, leading to the dissociation of Gα and Gβγ. Both Gα and Gβγ could activate their downstream targets like adenylate cyclases, phospholipases or protein kinases [6]. Gα subunits have been well characterized in many fungi, which were shown to engage in signal recognition, pathogenicity and infection structures, etc., in numerous plant pathogens [5,7,8].

In most characterized filamentous fungi, the Gα proteins are classified into three major groups including the group I (FadA, *A. nidulans*), group II (GanA, *A. nidulans*) and group III (GanB, *A. nidulans*) clans [6,9]. In filamentous fungi, the well-conserved group I Gα proteins are identical to human Gα_i_ superfamily proteins [6], which are involved in asexual development in fungal pathogenicity [10,11,12]. However, the fungal group II Gα proteins do not display a necessary function in fungal development, nor in their virulence processes among several fungal phytopathogens [10,11]. The fungal group III Gα proteins are highly conserved, as well, and many of them are important for regulation of cAMP synthesis [13,14,15]. The Gα subunit homologs have been well characterized in *Magnaporthe oryzae*, which possesses three Gα proteins (MagA, MagB and MagC), of which MagB plays an important role in fungal growth, conidiation and pathogenicity, and MagA has a limited role in conidiation, while MagA is dispensable for asexual development and appressorium formation [11]. In *Saccharomyces cerevisiae*, Gpa2 was shown to engage in fungal development by regulation of cAMP signaling [16]. A recent study in *Valsa mali* showed that the Gα proteins Gvm2 and Gvm3 regulated asexual development and fungal virulence via cAMP signaling [10]. The roles of Gα proteins have been studied in some *Aspergillus* species, most of which possess three Gα subunits [9,14,17,18]. In *A. nidulans*, GanB engaged in asexual development and spore germination [12], and FadA was shown to regulate sporulation and mycotoxin production [9]. The function of GanB homologue (GpaB) in *A. fumigatus* was also reported, which is important for conidia production and *A. fumigatus* virulence [19,20]. Studies in *Aspergillus* also demonstrated that Gα proteins affect catalase activity and proteinase production [17,18]. The Gα subunit homologs are also characterized in many other fungi, like the GNA-2 from *Neurospora crassa* [21] and FfG2/FfG3 from *Fusarium fujikuroi* [22].

We recently reported that the key modulators of cAMP, including the adenylate cyclase AcyA and the cAMP phosphodiesterase (hydrolyze cAMP) PdeH, regulated *A. flavus* development, virulence and AF biosynthesis [23,24]. In many fungal phytopathogens, signal transduction is critical for fungi in cross-talk with plants [10], so we queried if the upstream signal intermediator of cAMP, Gα subunits, were also important for AF biosynthesis and *A. flavus* pathogenicity. To explore the function of Gα subunits in *A. flavus*, we identified four Gα proteins, GpaB, GanA, FadA and GaoC, in this fungus and generated their deletion mutants. We then investigated the roles of one of the important Gα protein’s encoding gene on the growth, sporulation, sclerotia formation, AF production and virulence on crop seeds of *A. flavus* and demonstrated that *gpaB* was involved in modulating cAMP levels and PKA activities. Our main objective was to gain insight into the cross-talk between the upstream of cAMP signaling and the AF biosynthesis and pathogenicity attributes of *A. flavus*.

## 2. Results

### 2.1. Identification of G Protein α Subunits in Aspergillus flavus

**Figure 1:**
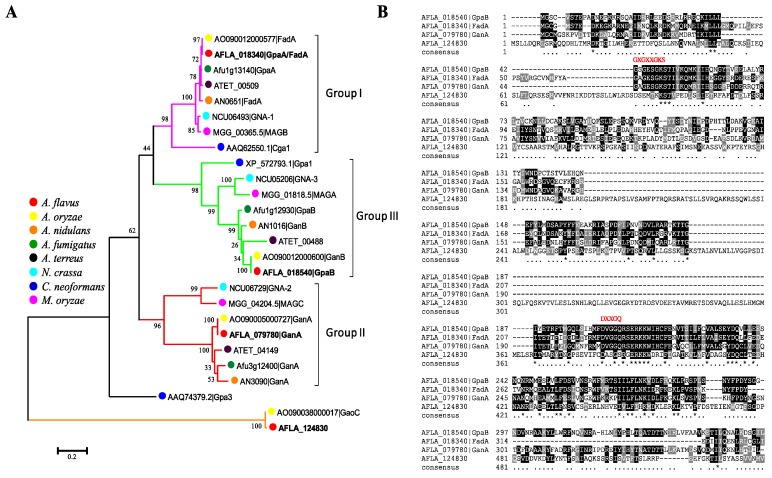
Characterization of G protein α subunits in *A. flavus*. (**A**) Phylogenetic analysis of the four *A*. *flavus* Gα proteins (FadA, GpaB, GanA and AFLA_124830) with characterized fungal Gα subunits. (**B**) Amino acid sequence alignments of the four Gα proteins (FadA, GpaB, GanA and AFLA_124830) of *A*. *flavus*. The conserved amino acid residues are shown in black, while the similar ones are shaded in gray.

Heterotrimeric G proteins (Gαβγ) play universal roles in signaling transduction in eukaryotes. Among the three G proteins subunits, Gα has been studied extensively and was shown to regulate multiple pathways. To identify the Gα proteins in *Aspergillus flavus*, the available protein sequences of the Gα from the selected *Aspergillus* species and other pathogenic fungi were downloaded from the NCBI database. The Gα sequences were used for phylogenetic analysis, which resolved into three main groups including the group I (FadA, *A. flavus*), group II (GanA, *A. flavus*) and group III (GpaB, *A. flavus*) clans (Figure 1A). Intriguingly, both *A. flavus* and *A. oryzae* possess an additional copy of the Gα subunit, GaoC, not found in other *Aspergillus* species (*A. nidulans*, *A. fumigatus* and *A. terreus*). The protein sequence analysis demonstrated that these Gα subunits only contain the unique G_alpha domain, which has GTPase activity and is responsible for binding guanyl nucleotide. FadA, GanA and GpaB in *A. flavus* all have the well-conserved GTP binding motif “GXGXXGKS” and GTPase domain “DXXXGQ” (Figure 1B), while the first three highly-conserved glycines (G) from the GTP binding motif in GaoC have been exchanged for aspartic acid (D), glutamic acid (E) and lysine (K), respectively, and the highly-conserved glutamine (Q) from the GTPase domain in GaoC has been exchanged for a serine residue (S) (Figure 1B). In addition, based on the prediction from SMART, although AFLA_124830 (*gaoC*) encodes a G_alpha domain, this domain is probably catalytically inactive.

### 2.2. Generation of Target Strains

**Figure 2:**
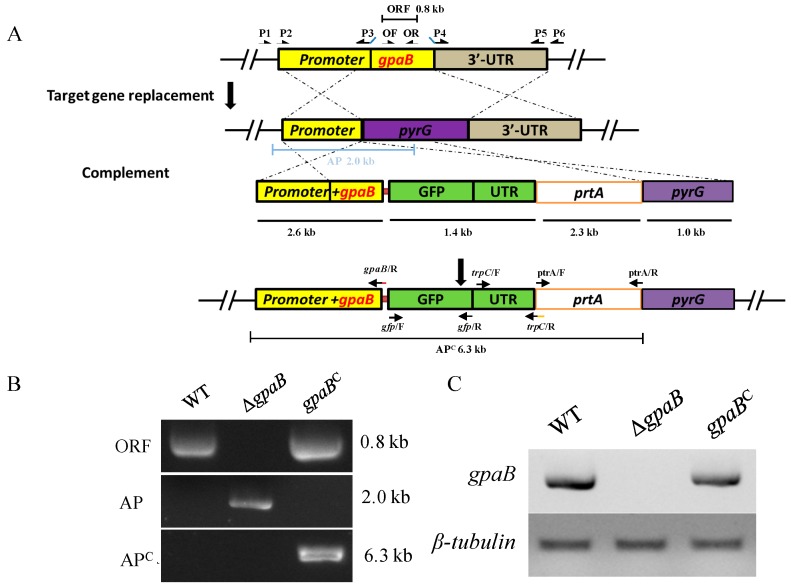
Deletion strategy and confirmation of the mutants used in this study. (**A**) Deletion and complement strategy for ∆*gpaB* strains; (**B**) Diagnostic PCR was performed to confirm the gene deletion and complemented strains. The *gpaB* ORF was confirmed by primers *gpaB*/OF and *gpaB*/OR; fragment AP was confirmed by primers *gpaB*/p1 and P801; while fragment AP^C^ was confirmed by primers *gpaB*/p1 and *ptrA/*R; (**C**) RT-PCR verification of *gpaB* gene deletion. The *β-tubulin* gene was used as a reference.

To understand the physiological function of the Gα subunit in *A. flavus*, the targeted gene replacement strategy was used to disrupt the Gα encoding genes from the CA14 PTs strain. Here, as GaoC was predicted dysfunctionally in *A. flavus*, we just generate the deficiency mutants of *ganA*, *gpaB* and *fadA*, but not for *gaoC*. The selected transformants were verified by diagnostic PCR and further confirmed by RT-PCR (Figure 2). However, we failed to obtain deletion strains for *fadA* from more than 200 ectopic transformants after many attempts, indicating that *fadA* might be important in this fungus, making it difficult to delete. Since the ∆*ganA* mutant displays a wildtype phenotype (data not shown), we did not generate a complementation strain for the ∆*ganA* mutant. The ∆*gpaB* mutant, which displayed an apparent phenotype defect compared to the wildtype *A. flavus*, was complemented with a wildtype gene copy fused with the *gfp* tag at its C-terminal and was confirmed both by diagnostic PCR and RT-PCR (Figure 2B,C). 

### 2.3. gpaB Is Involved in Pigmentation and Colonial Morphology in A. flavus

**Figure 3:**
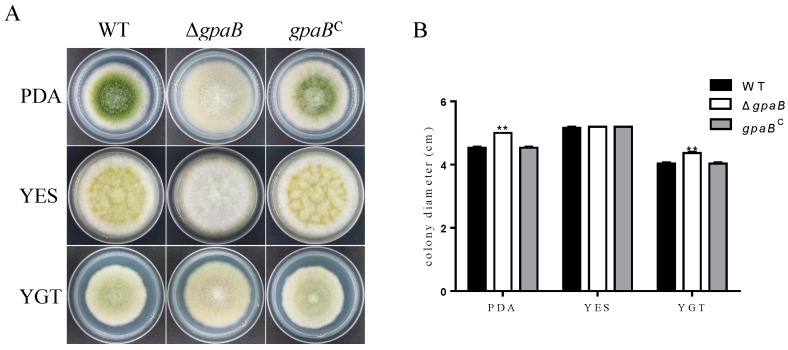
The Δ*gpaB* mutant was altered in pigmentation and vegetative growth. (**A**) Colony morphology of the WT, Δ*gpaB* and *gpaB^C^* strains after being grown on PDA, YGT and YES agar plates for four days at 37 °C. (**B**) Statistical analysis of the colony diameter of the indicated strains on different media. ** indicates significantly different between the wildtype and mutant strains (*p* ≤ 0.01), as assessed by one-way ANOVA and adjusted with Turkey’s multiple comparison test. The experiments were conducted with four replicates for the indicated strain and were repeated three times.

The colony size of the ∆*gpaB* mutant was increased compared with *A. flavus* wildtype (WT) and complemented strain (*gpaB^C^*) on PDA and YGT agar medium (Figure 3A,B). Furthermore, the ∆*gpaB* mutant produced white fluffy mycelium and no pigmentation compared with the somewhat woolly Kelly green colony of the WT and ∆*ganA* mutant (Figure 3A)*.* Importantly, the *gpaB^C^* complemented strain recovered all morphological defects (Figure 3A), indicating that the phenotypic alteration in the ∆*gpaB* mutant was caused directly by the disruption of the *gpaB* gene in *A. flavus*. These data indicate that *gpaB* is important for normal fungal morphology and pigmentation in *A. flavus*.

### 2.4. gpaB Is Required for Conidiation and Negatively Regulates Sclerotia Formation in A. flavus

**Figure 4:**
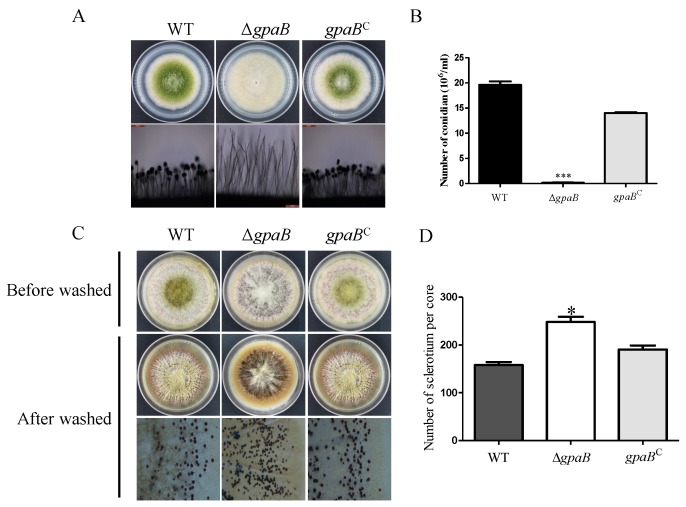
*gpaB* is involved in conidiation and sclerotia formation in *A*. *flavus*. (**A**) Colony and conidiophore morphology among the WT, Δ*gpaB* and *gpaB^C^* were observed after being grown on PDA agar medium for five days in the dark; (**B**) The number of conidia of the indicated strains was measured after being grown on PDA agar for five days; (**C**) Sclerotia formation of the indicated strains grown on sclerotia-inducing Wickerham (WKM) medium was detected. To visualize the sclerotia, 75% ethanol was sprayed on the WKM plates to remove the conidia; (**D**) The number of sclerotial was counted as in (**C**). Error bars represent the standard deviation from four replicates, and asterisks, “***” or “*”, represent significant differences compared to the wildtype according to the *t*-test with *p* < 0.001 and *p* < 0.05, respectively. The experiments were conducted with four replicates for the indicated strain and were repeated three times.

G protein signaling is important for asexual development in filamentous fungi. To know the role of *gpaB* functioning in *A. flavus* sporulation, the conidia production of WT, ∆*gpaB* and *gpaB^C^* strains was assayed after five days of growth on PDA agar media. As shown in Figure 4, deletion of *gpaB* led to a prominent decrease in conidiation in *A*. *flavus* (Figure 4A,B). The observation of aerial conidiophores also demonstrated that ∆*gpaB* failed to form normal conidiophore in ∆*gpaB* (Figure 4A). Further RT-qPCR analysis of conidia-specific genes’ expression showed that *brlA* and *abaA* were transcriptionally downregulated in ∆*gpaB* mutant compared to WT strains (*p* < 0.01) (Table 1). These data indicate that *gpaB* is essential for conidiation in *A*. *flavus*.

In addition to conidia, the sclerotium is another important reproductive structure for *A*. *flavus*. Our former study has shown that the cAMP signaling pathway might negatively regulate sclerotia development in *A*. *flavus*. To know if the Gα subunit GpaB is involved in sclerotia formation, we assayed the sclerotia reproduction in WT, ∆*gpaB* and *gpaB^C^* strains. The result showed that disruption of *gpaB* promoted sclerotia production compared to WT and *gpaB^C^* strains (*p* < 0.05) (Figure 4C,D). Additionally, the expression levels of sclerotia-related genes *nsdC*, *nsdD* and *sclR* were transcriptionally increased in the ∆*gpaB* mutant (*p* < 0.01) (Table 1). These results indicate that *gpaB* plays a negative role in sclerotial formation in *A. flavus*.

### 2.5. gpaB Is Involved in Stress Responses in A. flavus

**Figure 5:**
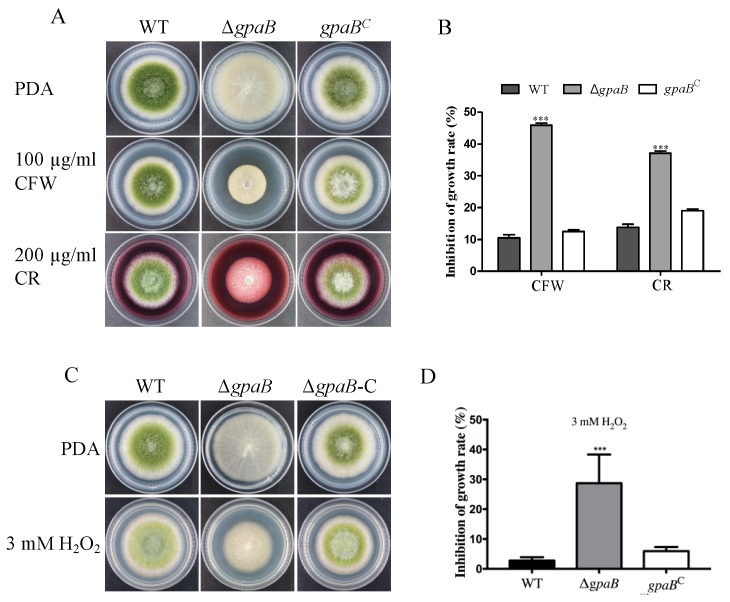
Δ*gpaB* is altered in its sensitivities to cell wall integrity stress and oxidative stress. (**A**) Colony phenotype of the indicated strains after being grown under cell wall integrity stress triggered by adding 100 µg/mL Calcofluor White (CFW) or 200 µg/mL Congo-Red (CR), for four days; (**B**) Statistical analysis of mycelia of the growth inhibition rate of the indicated strains under cell wall stress; (**C**) Colony phenotype of the indicated strains after being grown under oxidative stress triggered by 3 mM hydrogen peroxide (H_2_O_2_); (**D**) Statistical analysis of mycelia of the growth inhibition rate of the indicated strains under oxidative stress. Error bars represent the standard deviation from four replicates, and asterisks “***” represent significant differences compared to the wildtype according to the *t*-test with *p* < 0.001. The experiments were conducted with four replicates for the indicated strain and were repeated three times.

G protein signaling responds to multiple environmental signals including stress factors. To see the potential role of *gpaB* in stress response, the growth of the indicated strains was determined under different stress conditions. The results showed that the ∆*gpaB* mutant was much more sensitive to cell wall-damaging agent Calcofluor White (CFW) and Congo-Red (CR) than WT and *gpaB^C^* strains (*p* < 0.001) (Figure 5A,B). The ∆*gpaB* mutant also displayed less growth viability under oxidative stress generating by 3 mM hydrogen peroxide (H_2_O_2_) (*p* < 0.0001) (Figure 5C,D). These data indicate that *gpaB* is involved in stress responses in *A. flavus.*

### 2.6. gpaB Regulates AF Biosynthesis in A. flavus

**Figure 6:**
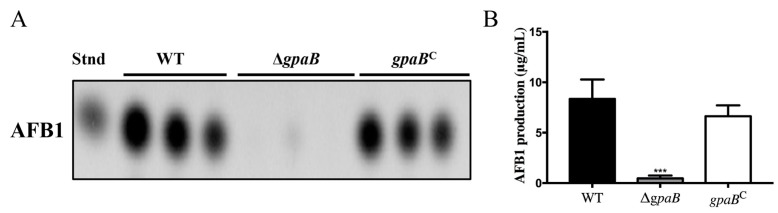
*gpaB* is required for AF biosynthesis. (**A**) AF production was measured by thin-layer chromatography (TLC) after being grown in YES medium in the dark at 29 °C for five days; (**B**) The amount of AF production in YES medium was qualified by Gene Tools analysis system software. The double asterisks “***” represent significant differences at *p* < 0.001. Stnd represents the AFB1 standard. The experiments were conducted with three replicates for the indicated strain and were repeated three times.

AFs are the most important secondary metabolites in *A*. *flavus*. Previous studies have demonstrated that G protein and its downstream cAMP signaling played a negative role in AF/sterigmatocystin (ST) biosynthesis in *A. nidulans*. Thus, we detected the AF production in the ∆*gpaB* mutant, and the result showed that AF biosynthesis was severely blocked in the ∆*gpaB* mutant compared to WT and *gpaB^C^* strains (*p* < 0.01) (Figure 6A,B). To further confirm that the loss production of AF in *gpaB*-deficient mutant was caused by the altered expression levels of AF biosynthesis genes, we performed RT-qPCR to analyze their expression levels, which demonstrated that the AF regulator coding genes *aflR* and *aflS* and its structure gene *aflP* were transcriptionally downregulated in the ∆*gpaB* mutant compared to WT strains (*p* < 0.001) (Table 1). All these results demonstrate that *gpaB* is important for AF biosynthesis in *A. flavus*.

### 2.7. gpaB Is Essential for A. flavus Pathogenicity on Maize Kernels

**Figure 7:**
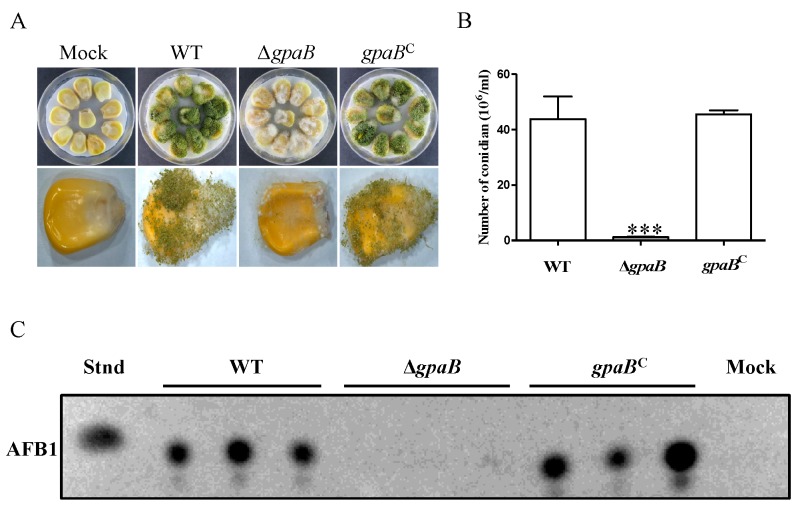
Disruption of *gpaB* leads to a significant reduction in seed infection. (**A**) Pathogenicity test of *gpaB* mutant on maize kernels; (**B**) Conidia production of *gpaB* mutant on maize kernels; (**C**) Detection of AF production of *gpaB* mutant on maize kernels by TLC. Error bars represent the standard deviation from three replicates, and triple asterisks “***” represent significant differences compared to the wildtype according to the *t*-test with *p* < 0.001. Stnd represents the AFB1 standard. The experiments were conducted with three replicates for the indicated strain and were repeated three times.

*A. flavus* contamination of many important seed crops has caused enormous economic losses. Here, we examined the *A*. *flavus* ∆*gpaB* mutant for their abilities to invade maize kernels. As shown in Figure 7, the ∆*gpaB* mutants grew less vigorously on maize seeds. A significant decrease in conidia production from the ∆*gpaB* mutant-infected maize kernels was also observed compared to the WT and *gpaB^C^* strains (*p* < 0.001) (Figure 7B). Additionally, AF biosynthesis from the ∆*gpaB* mutant-infected maize kernels was severely blocked (Figure 7C). All these data demonstrate that *gpaB* is important for *A. flavus* pathogenicity on maize seeds.

### 2.8. gpaB Regulates cAMP/PKA Signaling in A. flavus

**Figure 8:**
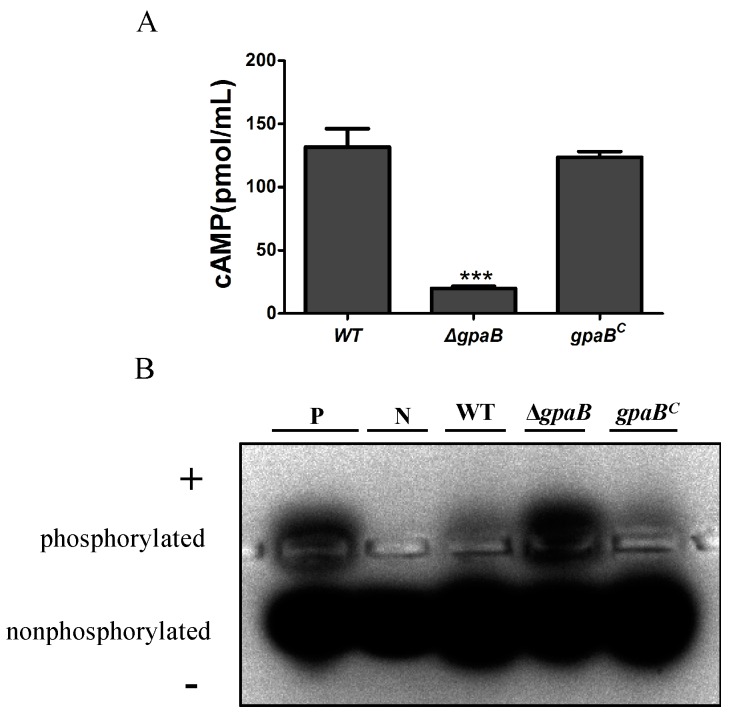
*gpaB* is involved in the regulation of intracellular cAMP levels and PKA activities in *A. flavus*. (**A**) Deletion of *gpaB* leads to decreased accumulation of cAMP levels in *A. flavus*; (**B**) Disruption of *gpaB* leads to a change of the cAMP-dependent protein kinase activities in *A. flavus*. A 0.8% agarose gel was used to separate protein samples. Phosphorylated peptides migrated towards the cathode (+), while nonphosphorylated peptides migrated towards the anode (−). P, phosphorylated sample control; N, non-phosphorylated sample control. The triple asterisks “***” represent significant differences compared to the wildtype according to the *t*-test with *p* < 0.001. The experiments were conducted with three replicates for the indicated strain and were repeated three times.

G proteins alpha subunits are shown to operate upstream of the cAMP signaling pathway, which could regulate the activity of adenylate cyclase. To determine whether *gpaB* is involved in cAMP synthesis in *A*. *flavus*, the intracellular cAMP levels were assayed after 48 h of inoculation, and we found that the ∆*gpaB* mutant demonstrated a prominent reduction in cAMP levels compared to the WT and *gpaB^C^* strains (*p* < 0.001) (Figure 8A). We further detected the effects of *gpaB* on cAMP-dependent protein kinase (PKA) activity; of interest, the ∆*gpaB* mutant, although showing downregulation of the intracellular cAMP levels, had a higher PKA activity compared to the WT and *gpaB^C^* strains (Figure 8B). Taken together, these results indicated that the G protein alpha subunit GpaB functions upstream of the cAMP signaling pathway activating cAMP synthesis and regulating PKA activities in *A. flavus*.

## 3. Discussion

The heterotrimeric G proteins are critical for signal transduction, which function in numerous biological processes, including vegetative growth, asexual and sexual development and fungal virulence from yeast to human or plant fungi [10,11,12,21,25]. G protein α subunits are the important upstream signal intermediator of cAMP signaling, for which we have recently demonstrated that it was involved in fungal development, pathogenicity and AF biosynthesis in *A. flavus* [23,24]. To know if Gα subunits are important for AF biosynthesis and *A. flavus* virulence, we here functionally characterized the Gα subunits GpaB, GanA and FadA in *A. flavus*. Intriguingly, the fourth Gα subunit GaoC was identified only in *Aspergillus flavus*/*oryzae* [26], while its potential role has not been studied yet. In *Ustilago maydis*, four distinct Gα subunits were characterized, as well, of which the novel Gpa4 has evolved dysfunctionally [27]. Here, we found that the G_alpha domain of GaoC in *A. flavus* was predicted to be probably inactive in *A. flavus*. In particular, substitutions of some conserved amino acid residues in the GTP binding motif were found in GaoC, which might affect the GTPase activity or the affinity for GDP/GTP binding.

Accumulating literature works indicated that Gα proteins play roles in regulating fungal asexual development. In *A. nidulans* and *A. fumigatus*, GanB/GpaB have been shown to be involved in conidia development and spore germination [12,19,20], and FadA was shown to regulate sporulation and mycotoxin production [9]. In *M. oryzae*, disruption of *magC* largely decreases spore production, while the *magA* deletion mutant does not display obvious defects in conidiation [11]. Here, we found that instead of *ganA*, *gpaB* plays an important role in regulation of asexual development. The ∆*gpaB* mutant displayed a defect in conidiophore formation and considerably reduced expression levels of conidia-specific genes *brlA* and *abaA*. These data indicate that the role of Gα subunits, especially for the group III Gα proteins, is quite conserved in fungi. In addition to conidiation [2], here, we also found that deletion of *gpaB* enhanced *A. flavus* sclerotia formation. Conidiation and sclerotia formation seem to remain balanced as reviewed previously. In this study, we also found that deleting the *gpaB* gene increased the transcript levels of sclerotia formation-related genes *sclR, nsdC* and *nsdD*, which might lead to abnormal sclerotia formation. All these indicated that GpaB is involved in the regulation of *A. flavus* asexual development. 

Many studies have shown that G proteins engage in fungal virulence [10,28]. In *A*. *fumigatus*, GapB was shown to engage in regulation of fungal infection [19,20]. The deletion of *gpaB* was almost avirulent in *A*. *fumigatus* [19]. Here, we found that deletion of *gpaB* causes a considerable reduction in the infection of crop seeds. In *A*. *flavus,* the pathogenesis is considered to be related to multiple factors, like sporulation, mycotoxins and adaptability to stress environments [1,2]. Here, we demonstrated that the ∆*gpaB* mutant grew less vigorously on maize kernels, which also severely impaired its sporulation on crop seeds. On the other hand, we found that the ∆*gpaB* mutant was defective in cell wall integrity, which might affect the colonization of *A. flavus* on crop seeds. In *Valsa mali*, the deletion of the Gα coding gene *gvm2*/*gvm3* showed a reduction in fungal virulence, which had a close relationship with the decreased transcriptional levels of cell wall-degrading enzyme genes [10]. The roles of group III Gα proteins on fungal virulence seem to be conserved in numerous plant pathogens, like *C. neoformans* (Gpa1), *Fusarium oxysporum* (Fga2) and *Botrytis cinerea* (Bcg3), for which the encoding gene is involved in the regulation of their pathogenesis [5,22,29]. 

In this study, we found that the deletion of *gpaB* led to a dramatic drop in the intracellular cAMP level, which was consistent with an *A. fumigatus* study showing that deletion of gpaB reduced the cAMP level in *A. fumigatus* [20], indicating that GpaB is important for the regulation of cAMP signaling in *A. flavus*. Previous studies in *A. nidulans*/*parasiticus* have demonstrated that the Gα protein FadA and its downstream cAMP signaling played a negative role in AF/ST biosynthesis [30,31]*.* Here, we found that the inactivation of GpaB blocked AF biosynthesis and its related genes’ expression, which might be caused by the decreasing level of intracellular cAMP in *A. flavus*. It is interesting to wonder why a downregulation of the intracellular cAMP levels by deleting *gpaB* from *A. flavus* results in a drop in AF biosynthesis and its regulated genes’ transcription levels. Of interest, we found that the full deletion of the *gpaB* gene showed a higher PKA activity compared to the WT and *gpaB^C^* strains, which might inhibit the activity of AF global transcription factor AflR and block AF production. On the other hand, we found that deletion of *gpaB* led to a prominent decrease in asexual development, which has a close relationship with AF biosynthesis. However, it is still hard to explicate why a decreased level of cAMP in the ∆*gpaB* mutant displays an increased activity of PKA. Our former study also indicated that abnormally high internal cAMP levels caused by the deletion of the phosphodiesterase encoding gene *pdes* decreased the PKA activities, and promoted AF production in *A. flavus* [24]. The dysfunction of GpaB might relieve GpaB-mediated repression of events leading to PKA activation. What also makes sense is that the Δ*gpaB* mutant showed a significant decrease in asexual sporulation, which is repressed by the activation of PKA in *A*. *flavus*.

## 4. Conclusions

In conclusion, four heterotrimeric Gα subunits were identified in *A. flavus*, and GpaB is important for asexual sporulation, AF biosynthesis and virulence by regulating cAMP signaling in *A. flavus*. These findings raise the possibility of designing specific strategies to prevent AF contamination and *A. flavus* invading important crops.

## 5. Materials and Methods

### 5.1. Strains and Culture Conditions

All strains utilized in this study are listed in Table 2. Potato dextrose agar (PDA, BD Difco, Franklin Lakes, NJ, USA) was used for the growth and conidiation assays, supplemented with the appropriate amounts of uridine (1 g/L), uracil (1 g/L) or pyrithiamine (0.1 μg/mL) when necessary. To analyze sclerotia production, the modified Wickerham medium (WKM) was used [32], and after being grown for 7 days, the sclerotium was visualized by using 70% ethanol to wash and kill conidia on the WKM plates. PDA agar supplemented with 100 μg/mL Calcofluor White (CFW), 200 μg/mL Congo-Red (CR) or 3 mM hydrogen peroxide (H_2_O_2_) was used to determine sensitivities to multiple stresses. The experiments were conducted with four replicates and repeated three times.

### 5.2. Strain Construction

The target gene deletion and transformation were conducted according to the previously described protocols [23]. For disruption of *gpaB* and *ganA*, a homologous recombination strategy was used to replace each gene with *Aspergillus fumigatus pyrG* in the parental strain *A*. *flavus* CA14 PTs protoplasts. The double-joint fusion PCR was performed to create the deletion constructs [34]. In brief, the flanking regions upstream and downstream of *gpaB* or *ganA* were amplified using primers P1 with P3 and P4 with P6 (Table 3), respectively. *A. fumigatus pyrG* was amplified from genomic DNA using primers *AfpyrG*/F and *AfpyrG*/R. The nested primers P2 and P5 were used to create entire disruption constructs (Table 3). The purified fusion PCR constructs were co-transformed into *A*. *flavus* CA14 PTs strain protoplasts.

To generate a *gpaB^C^* complemented strain, the *ptrA* selective marker was used. A 2.5-kb PCR product (1.1-kb *gpaB* coding sequence, 1.4-kb upstream sequence) was amplified from *A*. *flavus* wildtype genomic DNA using primers *gpaB*/P1 and *gpaB*/R (Table 3). A 0.7-kb *gfp* coding sequence and 0.7-kb *A*. *nidulans trpC* terminator region were amplified using primers *gfp*/F with *gfp*/R and *trpC*/F with *trpC*/R (Table 3), respectively, and a 1.0-kb 5′ flanking fragment of *A. fumigatus pyrG* was amplified using primers *AfpyrG*-cm/F and *AfpyrG*-cm/R. The *ptrA* marker was amplified from vector *pPTRI* (Takara, Tokyo, Japan) with primers *prtA*/F and *ptrA*/R. The primers *gpaB*/P2 and *AfpyrG*-cm-1049/R were used to generate the *gpaB* complemented construct containing the 2.5-kb *gpaB* complemented PCR product, *gfp* tag, trpC terminator, the *ptrA* selection marker and 5′ flanking region of the *AfpyrG* (Figure 2A). The purified fusion PCR construct was co-transformed into Δ*gpaB* strain protoplasts. The screened transformants were then confirmed by PCR and RT-PCR analysis.

### 5.3. Phylogenetic Analysis

For phylogenetic analysis, the G protein α subunit sequences of interest from the NCBI database (https://www.ncbi.nlm.nih.gov/protein/) were aligned together with *A*. *flavus* protein sequences (www.aspergillus.org) using MEGA5.1 software by the neighbor-joining method. Bootstrap analysis was performed with 1000 replicates.

### 5.4. AF Analysis

A 2.5 × 10^6^ spore suspension of *A. flavus* conidia was incubated into 25 mL YES medium in the dark at 29 °C for 5 days for AF production. AF extraction was performed according to the previously described protocol [35]. Then, thin layer chromatography (TLC) was used for AF production analysis. For quantitative analysis of AF production, Gene Tools image analysis software was used.

### 5.5. Fungal Virulence Assays on Corn

The fungal virulence was carried out following the previously described protocol [24,36,37]. The treated corn kernels were incubated with 200 μL 10^6^ conidia/mL of indicated strains in a 29 °C incubator under dark conditions for 5 d. The filter paper in the incubator was moistened every day. Three replicates were conducted for each strain. After the incubation, the infected kernels were collected in 50 mL Falcon tubes with 15 mL of sterile 0.05% Tween 80 water solution. One hundred microliters of spore suspension were removed for spore quantification. AF production from the infected kernels was extracted and analyzed following the previously described protocol [24,36,37].

### 5.6. Real-Time Quantitative Reverse Transcription PCR

Expression levels of genes involved in asexual development and AF biosynthesis were measured by RT-qPCR. The 48 h-old liquid shaken mycelium were harvested from PDA medium and lyophilized for the preparation of total RNA extraction. RNA was extracted from 100 mg of indicated mycelium using the Eastep Total RNA Extraction Kit (Promega, Madison, WI, USA) and treated with RNase-free DNase I (Thermo Fisher Scientific, Waltham, MA, USA). First-strand cDNA synthesis was performed using the RevertAid First Strand cDNA Synthesis Kit (Thermo Fisher Scientific, Waltham, MA, USA). To do RT-qPCR reaction, SYBR Green Supermix (Takara) was used and detected with the PikoReal 96 Real-time PCR system (Thermo Fisher Scientific, Waltham, MA, USA) using the program of an initial denaturing step at 95 °C for 5 min followed by 40 cycles, each consisting of denaturing at 95 °C for 5 s and extension at 60 °C for 30 s. The primers used for RT-qPCR are listed in Table 4. The efficiency of all the primers was between 90% and 110%.

### 5.7. Intracellular cAMP and PKA Activities Measurement

To assay the intracellular cAMP levels in the *A. flavus* strains, the liquid shaken mycelial were harvested after 48 h of inoculation and lyophilized overnight. The extraction of cAMP was conducted according to a previously-described method [24,38]. The Direct cAMP colorimetric (EIA) kit (Enzo Life Sciences, Exeter, U.K.) was used to quantify the cAMP levels. 

The free dry mycelium was used for total protein extraction following the protocol as previously described [24,39]. To measure the PKA activities, the PepTag^®^ Assay for Non-Radioactive Detection of the cAMP-Dependent Protein Kinase kit (Promega, Madison, WI, USA) was utilized.

### 5.8. Statistical Analysis

All data were presented as the means ± standard deviation (SD). GraphPad Prism 5 was used for statistical and significance analysis. The statistical differences were calculated using one-way ANOVA for multiple comparisons and adjusted with Turkey’s multiple comparison test. Student’s *t*-test was used when comparing two groups for differences. A *p*-value <0.05 was recognized as statistically significant. 

## Figures and Tables

**Table 1:** Expression levels of genes involved in asexual development and AF biosynthesis analyzed by RT-qPCR.

Gene ID	Name	Relative Expression Level	
		WT	Δ*gpaB*
*AFLA_029620*	*abaA*	1.00 ± 0.02	0.17 ± 0.01 **
*AFLA_082850*	*brlA*	1.00 ± 0.05	0.09 ± 0.01 **
*AFLA_131330*	*nsdC*	1.21 ± 0.13	4.28 ± 0.21 ***
*AFLA_020210*	*nsdD*	0.86 ± 0.05	2.91 ± 0.11 **
*AFLA_040260*	*sclR*	1.18 ± 0.18	7.20 ± 0.87 ***
*AFLA_139360*	*aflR*	0.88 ± 0.04	0.36 ± 0.03 ***
*AFLA_139340*	*aflS*	0.91 ± 0.09	0.26 ± 0.03 ***
*AFLA_139210*	*aflP*	1.11 ± 0.17	0.04 ± 0.0003 ***

Genes’ levels were normalized to the house keeping gene actin and calculated by the 2^−ΔΔCT^ method. Asterisks “**” and “***” represent significant differences compared to the wildtype for each gene according to the *t*-test with *p* < 0.01 and *p* < 0.001, respectively. The experiments were conducted with three replicates for the indicated strain and were repeated three times.

**Table 2:** *A*. *flavus* strains used in this study.

Strain	Genotype Description	Reference
*A. flavus* CA14 *PTs*	Δ*ku70*, Δ*pyrG*	[33]
wildtype	Δ*ku70,* Δ*pyrG*::*AfpyrG*	This study
Δ*gpaB*	Δ*ku70*, Δ*gpaB*::*AfpyrG*	This study
Δ*ganA*	Δ*ku70*, Δ*gpaB*::*AfpyrG*	This study
*gpaB^C^*	Δ*ku70*, Δ*gpaB*:: *AfpyrG*, *gpaB*(*p*):: *gpaB*::*gfp*::*ptrA*	This study

**Table 3:** Primers used for Gα genes’ deletion and complementation.

Primers	Sequence (5′-3′)	Application
*AfpyrG*/F	GCCTCAAACAATGCTCTTCACCC	*AfpyrG*
*AfpyrG*/R	GTCTGAGAGGAGGCACTGATGC	
P801/R	CAGGAGTTCTCGGGTTGTCG	
*gpaB*/P1	ACGAGTAACACCCTGTGAATGG	*gpaB* deletion flanking regions
*gpaB*/P3	GGGTGAAGAGCATTGTTTGAGGCGAACGGCATCAACCTATCACG	
*gpaB*/P4	GCATCAGTGCCTCCTCTCAGACTGAGACTTTGTGGCATGGAGT	
*gpaB*/P6	GTCCAGACCCTTCCTACAACTC	
*gpaB*/P2	TTACCGTCACAACCCTTCAGC	*gpaB* deletion construct nest primers
*gpaB*/P5	GTCCAGACCCTTCCTACAACTC	
*gpaB/*OF	GCCTATCACCAGTTCTCCCTT	*gpaB* mutant screen
*gpaB/*OR	CACATCATTGCCGCCAGAG	
*ganA*/P1	CGTACTCGTTCCCTACTGACAG	*ganA* deletion flanking regions
*ganA*/P3	GGGTGAAGAGCATTGTTTGAGGCAGTCCAATGGCAGCAGGTG	
*ganA*/P4	GCATCAGTGCCTCCTCTCAGACCCTTTCTACGACACTTTGGC	
*ganA*/P6	AAGGTGTTGGGTGGAGGGA	
*ganA*/P2	TCGTTGTCGCTTACCTACTGC	*ganA* deletion construct nest primers
*ganA*/P5	CTCCTTCCGCATTAGACACC	
*ganA/*OF	CAAGTTTATCTCGGCAATGTG	*ganA* mutant screen
*ganA/*OR	CTCCACTAAGCACTGGTCGT	
*gpaB*/R	GGCTCCAGCGCCTGCACCAGCTCCCAAGATACCTGAATCCTTCAAAG	*gpaB* mutant complement
*AfpyrG*-cm/F	GGATCCCGTAATCAATTGCCCATTGCCTCAAACAATGCTCTTCACCC	
*ptrA*/F	AATGGGGTGACGATGAGCC	*ptrA*
*ptrA*/R	AATGGGCAATTGATTACGGG	
*gfp*/F	GGAGCTGGTGCAGGCGCTGGAGCCGGTGCCATGGTGAGCAAGGGCGAGGA	*GFP*
*gfp*/R	TCAAAGATCTACCATGTACAGC	
*trpC*/F	TTGATGATTTCAGTAACGTTAATTAACGTTACTGAAATCATCAA	*trpC* terminator
*trpC*/R	AAGAGCGGCTCATCGTCACCCCATTAAGAAGGATTACCTCTAAACAA	

**Table 4:** Primers used for RT-qPCR.

Primers	Sequence (5′-3′)	Length	Application
*actin-F*	ACGGTGTCGTCACAAACTGG	129 bp	RT-PCR for *actin*
*actin-R*	CGGTTGGACTTAGGGTTGATAG		
*abaA-F*	TCTTCGGTTGATGGATGATTTC	84 bp	RT-PCR for *abaA*
*abaA-R*	CCGTTGGGAGGCTGGGT		
*brlA-F*	GCCTCCAGCGTCAACCTTC	158 bp	RT-PCR for *brlA*
*brlA-R*	TCTCTTCAAATGCTCTTGCCTC		
*nsdC-F*	GCCAGACTTGCCAATCAC	153 bp	RT-PCR for *nsdC*
*nsdC-R*	CATCCACCTTGCCCTTTA		
*nsdD-F*	GGACTTGCGGGTCGTGCTA	167 bp	RT-PCR for *nsdD*
*nsdD-R*	AGAACGCTGGGTCTGGTGC		
*sclR-F*	CAATGAGCCTATGGGAGTGG	102 bp	RT-PCR for *sclR*
*sclR-R*	ATCTTCGCCCGAGTGGTT		
*aflR-F*	AAAGCACCCTGTCTTCCCTAAC	233 bp	RT-PCR for *aflR*
*aflR-R*	GAAGAGGTGGGTCAGTGTTTGTAG		
*aflS-F*	CGAGTCGCTCAGGCGCTCAA	134 bp	RT-PCR for *aflS*
*aflS-R*	GCTCAGACTGACCGCCGCTC		
*aflP-F*	GATTGGGATGTGGTCATGCGATT	181 bp	RT-PCR for *aflP*
*aflP-R*	GCCTGGGTCCGAAGAATGC		
